# Supplementary material for: Equine Incisor Lesions: Histologic Confirmation of Radiographic, Macroscopic, and Micro-Computed Tomographic Findings
Source: Vet Sci. 2022 Jul 11;9(7):348. doi: 10.3390/vetsci9070348 (PMC9323279; doi:10.3390/vetsci9070348)
Supplement: Supplementary file 1 [file vetsci-09-00348-s001.zip › vetsci-1774831-supplementary.pdf]

Supplement Table S1: Detailed Distribution of teeth in the five categories.

			All teeth	Age Group (I)	Age Group (II)	Age Group (III)
**Category 1**	radiographically<br>macroscopically<br>µCT	healthy<br>healthy<br>healthy	19 (10.2%)	17 (53.1%)	0 (0.0 %)	2 (2.4%)
**Category 2**	radiographically<br>macroscopically<br>µCT	healthy<br>healthy<br>suspicious	26 (30.5%)	10 (71.4%)	6 (16.7%)	9 (25.7%)
	radiographically<br>macroscopically<br>µCT	healthy<br>healthy<br>moderate	30 (35.3%)	4 (28.6%)	8 (22.2%)	18 (51.4%)
	radiographically<br>macroscopically<br>µCT	healthy<br>suspicious<br>healthy	2 (2.4%)	0 (0.0 %)	2 (5.5%)	0 (0.0 %)
	radiographically<br>macroscopically<br>µCT	healthy<br>suspicious<br>suspicious	6 (7.1%)	0 (0.0 %)	6 (16.7%)	1 (2.9%)
	radiographically<br>macroscopically<br>µCT	healthy<br>suspicious<br>moderate	20 (23.5%)	0 (0.0 %)	13 (36.1%)	7 (20.0%)
	radiographically<br>macroscopically<br>µCT	healthy<br>moderate<br>suspicious	1 (1.2%)	0 (0.0 %)	1 (2.8%)	0 (0.0 %)
			85 (100%)	14 (100%)	36 (100%)	35 (100%)
**Category 3**	radiographically<br>macroscopically<br>µCT	healthy<br>moderate<br>moderate	17 (68.0%)	0 (0.0 %)	10 (62.5%)	7 (77.8%)
	radiographically<br>macroscopically<br>µCT	healthy<br>moderate<br>severe	3 (12.0%)	0 (0.0 %)	2 (12.5%)	1 (11.1%)
	radiographically<br>macroscopically<br>µCT	healthy<br>severe<br>moderate	1 (4.0%)	0 (0.0 %)	1 (6.25%)	0 (0.0 %)
	radiographically<br>macroscopically<br>µCT	healthy<br>severe<br>severe	1 (4.0%)	0 (0.0 %)	1 (6.25%)	0 (0.0 %)
	radiographically<br>macroscopically<br>µCT	healthy<br>suspicious<br>severe	3 (12.0%)	0 (0.0 %)	2 (12.5%)	1 (11.1%)
			25 (100%)	0 (0.0 %)	16 (100%)	9 (100%)

Category 4	radiographically	suspicious				
	macroscopically	healthy	1 (3.7%)	1 (100%)	0 (0.0 %)	0 (0.0 %)
	µCT	suspicious				
	radiographically	suspicious				
	macroscopically	healthy	6 (22.2%)	0 (0.0 %)	3 (33.3%)	3 (17.6%)
	µCT	moderate				
	radiographically	suspicious				
	macroscopically	suspicious	1 (3.7%)	0 (0.0 %)	0 (0.0 %)	1 (5.9%)
	µCT	healthy				
	radiographically	suspicious				
	macroscopically	suspicious	1 (3.7%)	0 (0.0 %)	0 (0.0 %)	1 (5.9%)
	µCT	suspicious				
	radiographically	suspicious				
	macroscopically	suspicious	6 (22.2%)	0 (0.0 %)	5 (55.6%)	1 (5.9%)
	µCT	moderate				
	radiographically	suspicious				
	macroscopically	moderate	8 (29.7%)	0 (0.0 %)	1 (11.1%)	7 (41.2%)
	µCT	moderate				
	radiographically	moderate				
	macroscopically	moderate	4 (14.8%)	0 (0.0 %)	0 (0.0 %)	4 (23.5%)
	µCT	moderate				
			27 (100%)	1 (100%)	9 (100%)	17 (100%)
Category 5	radiographically	suspicious				
	macroscopically	suspicious	5 (16.7%)	0 (0.0 %)	3 (33.3%)	2 (9.5%)
	µCT	severe				
	radiographically	suspicious				
	macroscopically	moderate	5 (16.7%)	0 (0.0 %)	4 (44.5%)	1 (4.8%)
	µCT	severe				
	radiographically	suspicious				
	macroscopically	severe	1 (3.3%)	0 (0.0 %)	1 (11.1%)	0 (0.0 %)
	µCT	moderate				
	radiographically	suspicious				
	macroscopically	severe	2 (6.7%)	0 (0.0 %)	1 (11.1%)	1 (4.8%)
	µCT	severe				
	radiographically	moderate				
	macroscopically	moderate	5 (16.7%)	0 (0.0 %)	0 (0.0 %)	5 (23.8%)
	µCT	severe				
	radiographically	moderate				
	macroscopically	severe	4 (13.2%)	0 (0.0 %)	0 (0.0 %)	4 (19.0%)
	µCT	moderate				
	radiographically	moderate				
	macroscopically	severe	5 (16.7%)	0 (0.0 %)	0 (0.0 %)	5 (23.8%)

	μCT	severe				
	radiographically<br>macroscopically<br>μCT	severe<br>severe<br>moderate	1 (3.3%)	0 (0.0 %)	0 (0.0 %)	1 (4.8%)
	radiographically<br>macroscopically<br>μCT	severe<br>severe<br>severe	2 (6.7%)	0 (0.0 %)	0 (0.0 %)	2 (9.5%)
			30 (100%)	0 (0.0 %)	9 (100%)	21 (100%)

Supplement Table S2: Detailed categorisation of histologically examined teeth.

Tooth (n=18)	Age Group	Clinical assessment*	Category	Radiographic assessment	Macroscopic assessment	µCT assessment	Stereomicroscopic assessment	Histologic assessment
7_101	(I)	healthy	1	healthy	healthy	healthy	suspicious	suspicious
12_403	(I)	healthy	1 / '0'	healthy	healthy	healthy	healthy	healthy
15_301	(I)	healthy	1 / '0'	healthy	healthy	healthy	healthy	healthy
19_203	(I)	healthy	1 / '0'	healthy	healthy	healthy	healthy	healthy
6_202	(II)	EOTRH	2	healthy	suspicious	suspicious	suspicious	suspicious
8_301	(II)	suspicious	-	healthy	healthy	-	healthy	suspicious
11_202	(II)	suspicious	4	suspicious	healthy	moderate	healthy	moderate
11_402	(II)	suspicious	4	suspicious	moderate	moderate	healthy	moderate
16_102	(II)	healthy	-	healthy	healthy	-	suspicious	healthy
3_302	(III)	suspicious	2	healthy	healthy	moderate	suspicious	suspicious
9_401	(III)	suspicious	4	suspicious	suspicious	moderate	healthy	moderate
10_101	(III)	EOTRH	3	healthy	moderate	moderate	moderate	severe
10_202	(III)		5	moderate	severe	severe	moderate	moderate
14_102	(III)	EOTRH	5	moderate	moderate	severe	moderate	moderate
14_202	(III)		5	moderate	moderate	severe	severe	severe
14_402	(III)		5	moderate	severe	severe	severe	severe
18_103	(III)	EOTRH	5	moderate	severe	moderate	severe	severe
18_203	(III)		5	moderate	severe	severe	severe	severe

* The clinical scoring was based on findings of the whole incisor region, not on a single tooth basis.
